# Supplementary material for: Nrf1 promotes heart regeneration and repair by regulating proteostasis and redox balance
Source: Nat Commun. 2021 Sep 6;12:5270. doi: 10.1038/s41467-021-25653-w (PMC8421386; doi:10.1038/s41467-021-25653-w)
Supplement: Supplementary file 1 — Supplementary Information [file 41467_2021_25653_MOESM1_ESM.pdf]

SUPPLEMENTARY INFORMATION:

Nrf1 promotes heart regeneration and repair by regulating proteostasis and redox balance

Miao Cui[12], Ayhan Atmanli[12], Maria Gabriela Morales[12], Wei Tan[12], Kenian Chen[3], Xue Xiao[3], Lin Xu[3], Ning Liu[12], Rhonda Bassel-Duby[12], and Eric N. Olson[12*]

[1]Department of Molecular Biology, the Hamon Center for Regenerative Science and Medicine, University of Texas Southwestern Medical Center, Dallas, USA

[2]Senator Paul D. Wellstone Muscular Dystrophy Specialized Research Center, University of Texas Southwestern Medical Center, Dallas, USA

[3]Quantitative Biomedical Research Center, Department of Population & Data Sciences and Department of Pediatrics, University of Texas Southwestern Medical Center, Dallas, USA

*Correspondence:

Eric N. Olson, Ph.D.

5323 Harry Hines Boulevard, Dallas, Texas, 75390-9148

Tel: 214-648-1187 E-mail: eric.olson@utsouthwestern.edu

Supplementary Figures 1-15

Supplementary Table 1

a

Samples	Number of spots	Mean mapped reads per spot	Median gene per spot	Sequencing saturation	Genome mapping rate	Reads mapped to transcriptome	Fraction reads in spot	Total genes detected
P1-Sham-d3	544	188,589	4,854	85%	95%	86%	86%	16,899
P1-MI-d3	376	279,464	6,322	83%	94%	85%	85%	17,615
P1-Sham-d7	960	106,531	4,877	71%	94%	86%	89%	17,643
P1-MI-d7_rep1	693	181,925	7,024	61%	92%	81%	78%	18,300
P1-MI-d7_rep2	791	145,276	4,666	80%	95%	87%	85%	17,601

b

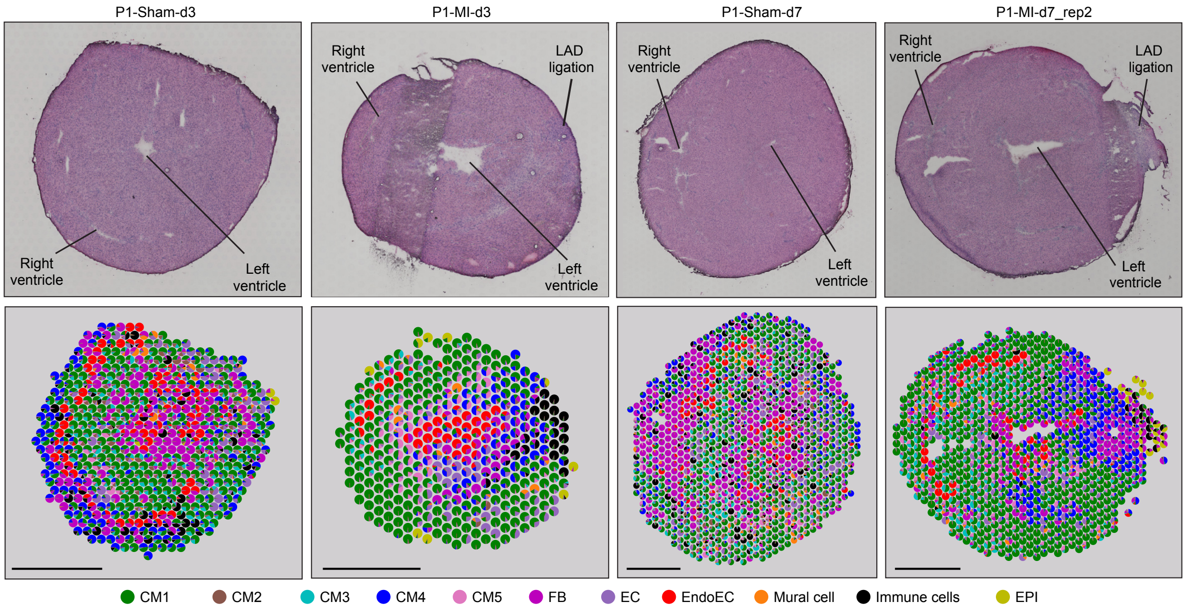

c

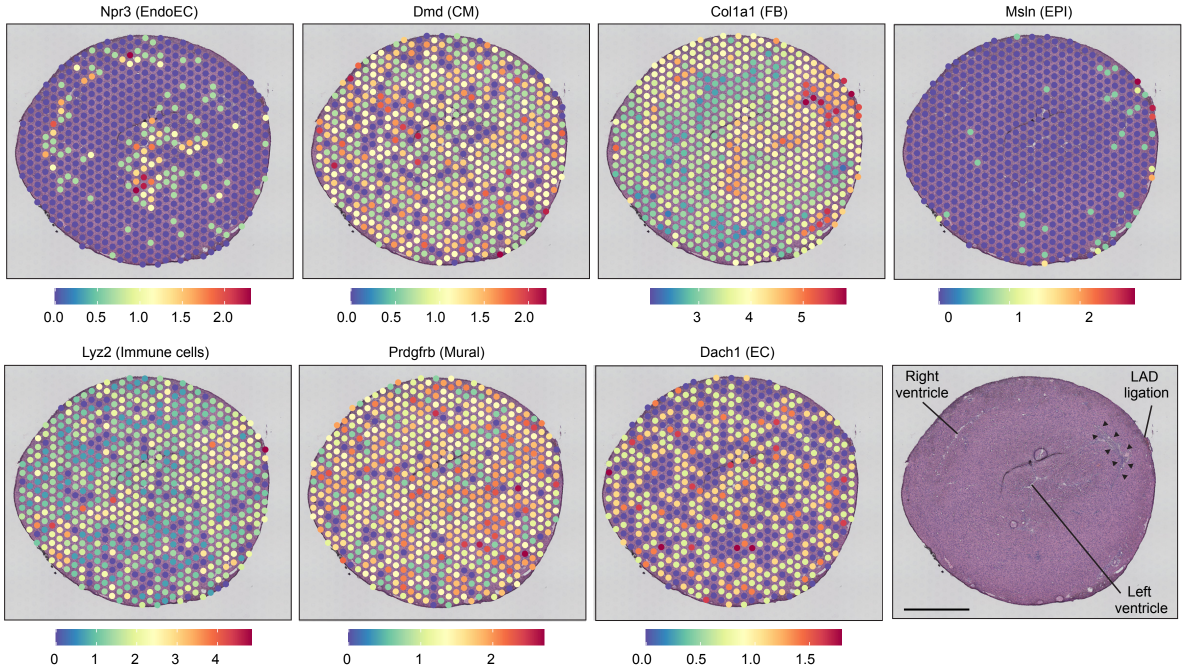

Supplemental Figure 1

Supplementary Fig. 1. Spatial transcriptional analysis of the regenerating heart identifies anatomic localization of cardiac cell types. a, Sequencing statistics of each sample. **b,** Upper panels: H&E staining of heart sections at indicated stage; lower panels: cell type composition shown in pie-charts for individual spatial spot mapped onto heart sections; Scale bar, 500 μm. **c**, Spatial expression of marker genes for endocardial cells (EndoEC), cardiomyocytes (CM), fibroblasts (FB), epicardial cells (EPI), immune cells, Mural cells, and endothelial cells (EC) on a heart section collected at 7-day post P1 MI with H&E staining shown on lower right (black arrowheads depict fibrotic tissue); Scale bar, 500um; **b, c**, for samples P1-Sham-d3, P1-MI-d3, and P1-Sham-d7, experiments were performed on one biological sample; sample P1-MI-d7_rep2 is an independent repeat of P1-MI-d7_rep1 (Fig.1), which showed similar results.

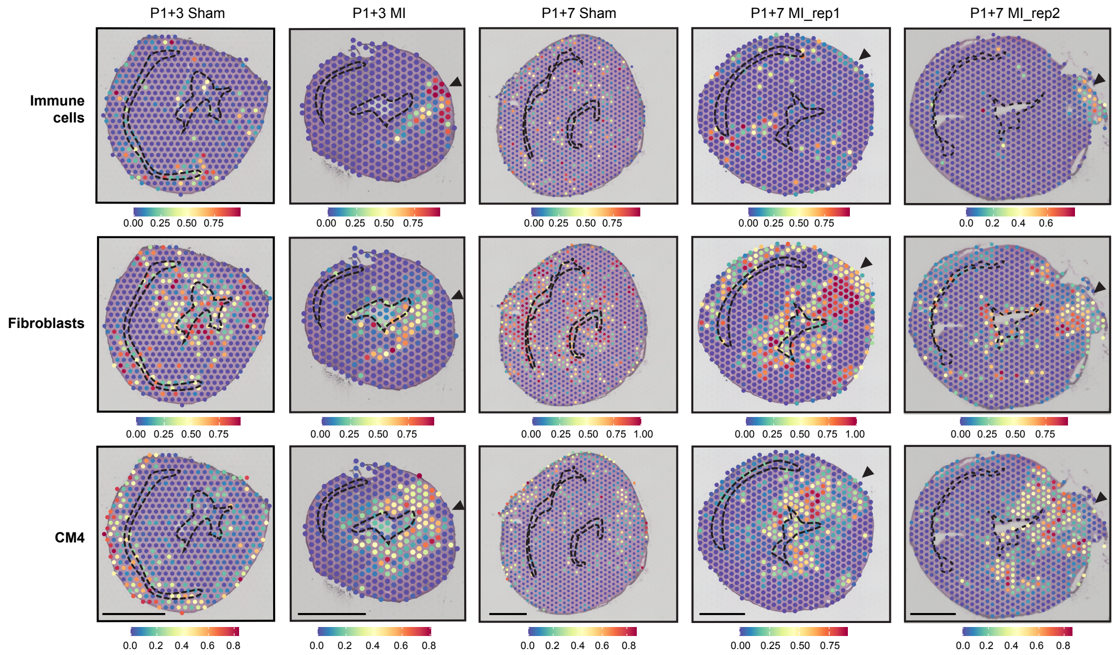

Supplementary Fig. 2. Dynamic localization of immune cells, fibroblasts, and CM4 cells during heart regeneration. Anatomic localization of immune cells, fibroblasts, and CM4 on heart sections collected at 3-day and 7-day post- Sham or MI surgery. Endocardium is outlined by black dash lines; injury site is marked by arrowhead; Scale bar, 500 µm. For samples P1-Sham-d3, P1-MI-d3, and P1-Sham-d7, experiments were performed on one biological sample. Samples P1-MI-d7_rep2 and P1-MI-d7_rep1 are two biologically independent replicates with similar results.

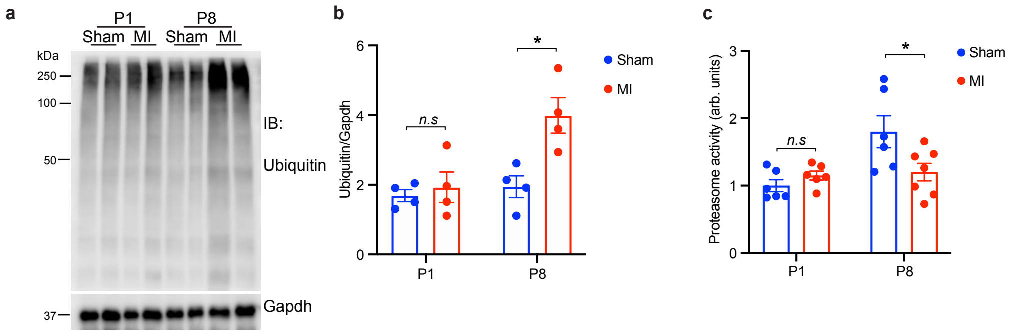

Supplementary Fig. 3. Maintained proteasomal activity in P1 regenerative heart after MI. a, Western blot analysis showing accumulation of poly-ubiquitinated proteins in P8 hearts, but not P1 hearts, at 1-day after MI compared to Sham surgery. **b**, Quantification showing normalized ubiquitin protein levels in (**a**); n=4 animals for each group derived from two experiments. Samples derived from the same experiment and Western blots used for quantification were processed in parallel, *p=0.0427 by Student's t-test two-tailed. **c**, Chymotrypsin-like activity of the 20S proteasome in P1 and P8 hearts at 1-day post-MI or Sham measured by substrate cleavage assay in cell lysate; n=5 animals for group P1-MI and n=6 animals for each of the rest groups, *p=0.0417 by Student's t-test two-tailed. **b, c**, results are shown as mean ± s.e.m; *n.s*. not significant; arb. units, arbitrary units.

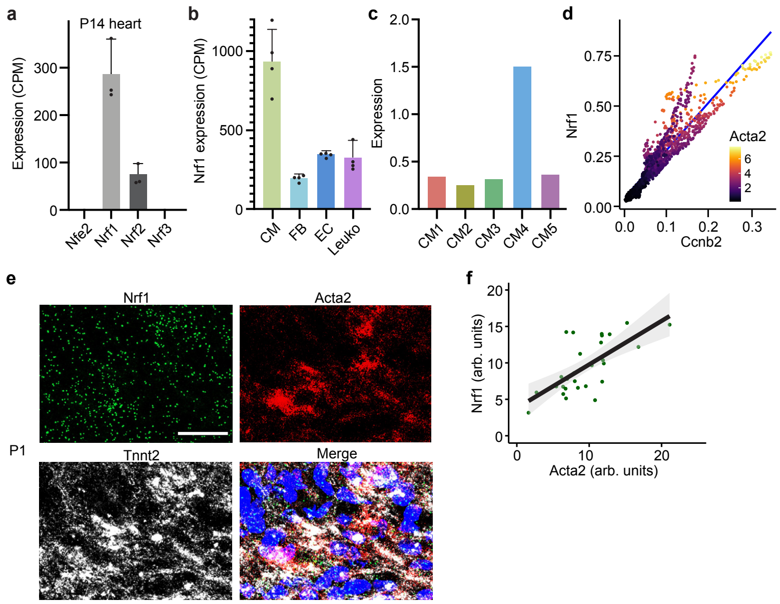

Supplementary Fig. 4. *Nrf1* expression in the heart. a, Expression shown as counts per million reads (CPM) of Nfe2 family genes (*Nfe2*, *Nrf1*, *Nrf2*, and *Nrf3*) in P14 mouse hearts; n=3 biologically independent animals. **b**, Expression shown as counts per million reads (CPM) of *Nrf1* in cardiomyocytes (CM), fibroblasts (FB), endothelial cells (EC) and leukocytes (Leuko) of P56 mouse heart; n=4 biologically independent animals. Data from GSE95755. **c**, Averaged expression of *Nrf1* in CM1-CM5 cells from our previous snRNA-seq data (GSE130699). Expression levels are shown as reads per 10k counts. **d,** Expression correlation plots between *Nrf1*, *Ccnb2*, and *Acta2* in individual cardiomyocytes from our previous snRNA-seq data (GSE130699). *Nrf1* positively correlates with *Ccnb2* (Spearman's coefficient=0.91). Expression levels are shown as reads per 10k counts. **e**, *Nrf1*, *Acta2*, and *Tnnt2* RNA transcripts detected by RNA-scope probes in the left ventricular myocardium on a transverse section of P1 hearts. Scale bar, 20 μm. **f**, Quantification of *Nrf1* and *Acta2* transcript levels in cardiomyocytes from **e**; n=29-34 cardiomyocytes were quantified. *Nrf1* expression level positively correlates with *Acta2*

(Spearman's coefficient=0.65); shaded area represents 95% Confidence Interval of the regression line. **a**,**b**, Results are shown as mean ± s.e.m; arb. units. arbitrary units.

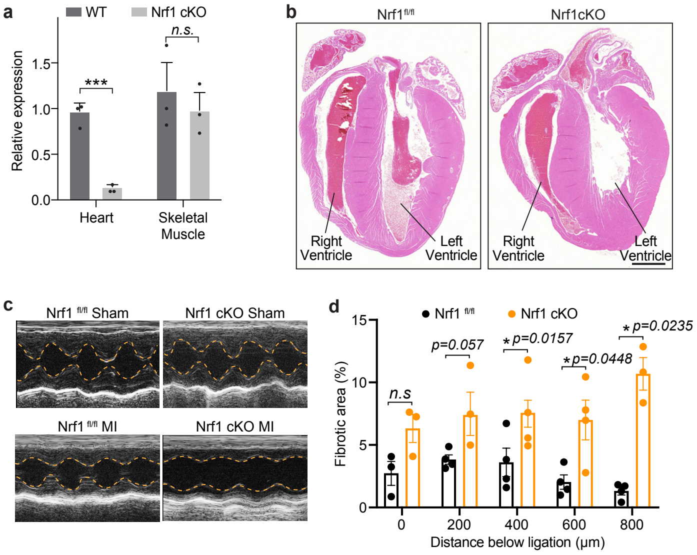

Supplementary Fig. 5. Cardiomyocyte-specific knockout of Nrf1 impairs neonatal heart regeneration. a, *Nrf1* expression (qPCR) in hearts and skeletal muscle of Nrf1 cKO and WT mice at P14; n=3 animals for each group. Results are shown as mean ± s.e.m. ***p=0.0009, Student's t-test two-tailed. **b**, H&E staining of heart sections from 2-month-old Nrf1 cKO and Nrf1$^{fl/fl}$ mice. Scale bar, 1000 μm; experiments were repeated three times with similar results. **c**, Myocardial wall motion (highlighted in orange) in Nrf1$^{fl/fl}$ and Nrf1 cKO mouse hearts at day-25 after MI or Sham surgery performed at P3; experiments were repeated three times with similar results. **d**, Quantification of fibrotic area at multiple plane levels below the ligation in Nrf1$^{fl/fl}$ and Nrf1 cKO mouse hearts at day-25 post-MI or Sham surgery performed at P3; n=4 animals for each group. Results are shown as mean ± s.e.m. *p<0.05 with exact p-value depicted, Student's t-test two-tailed.

a

Samples	Number of cardiomyocytes	Mean mapped reads per cell	Median gene per cell	Genome mapping rate	Reads mapped to transcriptome	Fraction reads in cells	Total genes detected
WT-MI	2,003	15,973	1,564	95%	79%	54%	23,995
WT-Sham	1,406	15,160	1,211	95%	88%	53%	23,427
cKO-MI	1,848	17,263	1,559	94%	79%	51%	23,758
cKO-Sham	3,328	10,044	1,258	95%	81%	56%	23,495

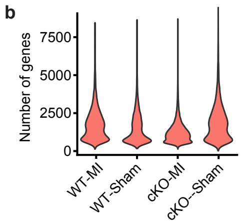

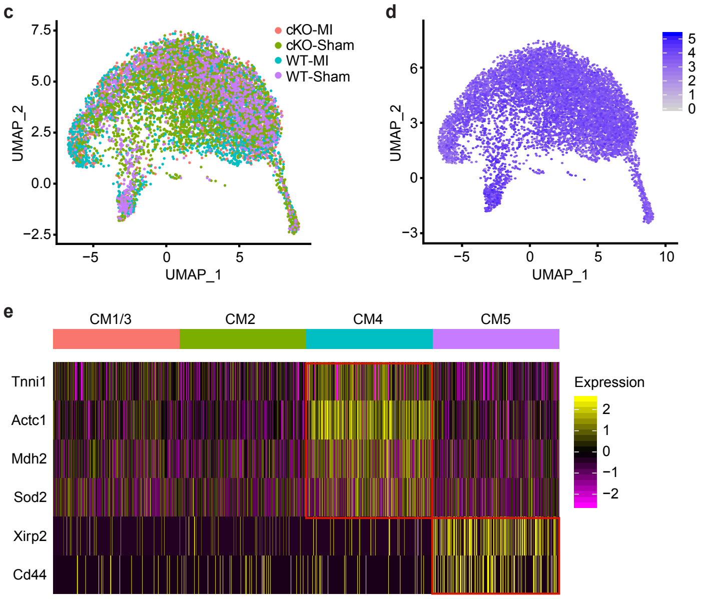

Supplementary Fig. 6. snRNA-seq analysis identifies cardiomyocyte subpopulations in Nrf1 cKO mouse hearts. a, Sequencing statistics of each sample**. b,** Violin plots showing the distribution of detected genes in each sample. **c,** UMAP visualization of cardiomyocyte clusters colored by sample of origin. **d,** Heatmap of *Tnnt2* expression in each cardiomyocyte cluster projected on UMAP graph. Color scale represents the expression of *Tnnt2*. **e**, Heatmap showing the expression of representative CM4 markers and CM5 markers in CM1/3, CM2, CM4, CM5 cells.

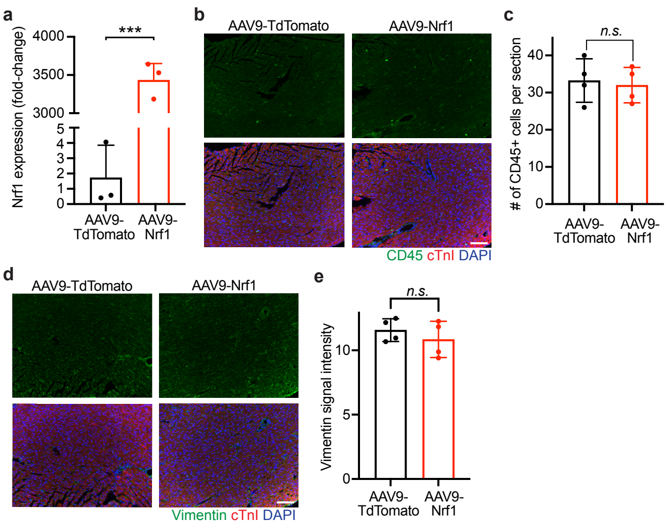

Supplementary Fig. 7. AAV9-mediated Nrf1 overexpression in adult hearts. a, qPCR measurement of *Nrf1* expression in mouse hearts 8-weeks after AAV9-TdTomato and AAV9-Nrf1 injection at P4; n=3 for each group; ***p<0.0001. **b,** Immunostaining on heart sections collected from uninjured adult mice showing comparable levels of tissue-resident immune cells (marked by CD45) in AAV9-TdTomato and AAV9-Nrf1 hearts. **c,** Quantification of $CD45^{+}$ cells. n=2 mice for each group; each mouse is counted in two replicate sections. **d,** Immunostaining on heart sections collected from uninjured adult mice showing similar frequency of cardiac fibroblasts ($Vimentin^{+}$) in AAV9-TdTomato and AAV9-Nrf1 hearts. **e,** Quantification of fibroblasts, measured as signal intensity of Vimentin staining. n=2 mice for each group; each mouse is counted in two replicate sections. Scale bar, 100 μm. **a**, **c**, **e**, results are shown as mean ± s.d; Student's t-test two-tailed**;** *n.s.* not significant

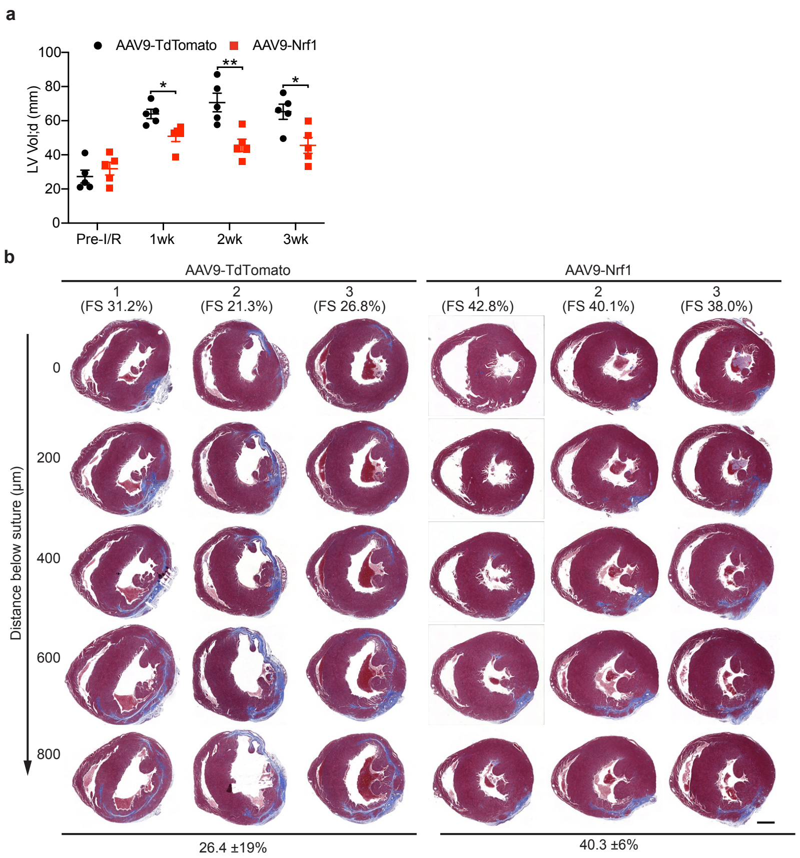

Supplementary Fig. 8. Nrf1 confers protection in adult mouse heart after I/R. a, Diastolic volume of left ventricles from AAV-TdTomato and AAV-Nrf1 hearts before I/R (Pre-I/R), and 1, 2, and 3 weeks (wk) after I/R; n=5 animals for each group. 1wk, *p=0.0129; 2wk, **p=0.005; 3wk, *p=0.015; results are shown as mean ± s.d; Student's t-test two-tailed**. b**, Masson's trichrome staining showing fibrotic scarring on transverse sections of hearts from mice injected with AAV9-TdTomato or AAV9-Nrf1. Samples were collected at 3 weeks after I/R. Images of heart sections at 0, 200, 400, 600, and 800 µm below the ligation point are depicted. Fractional shortening (FS) values for corresponding hearts are depicted. Scale bar, 500 µm.

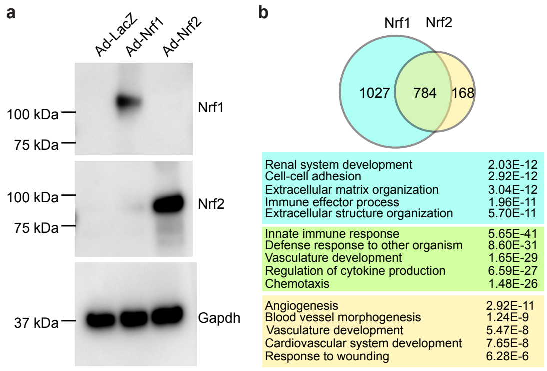

Supplementary Fig. 9. Nrf1 confers greater protection than Nrf2. a, Western blot analysis showing overexpression of Nrf1 and Nrf2 using adenoviral (Ad) expression in NRVMs; experiments were repeated twice with similar results. **b**, Venn diagram showing number of genes downregulated by Nrf1, Nrf2, or by both (top) and their associated GO terms (bottom).

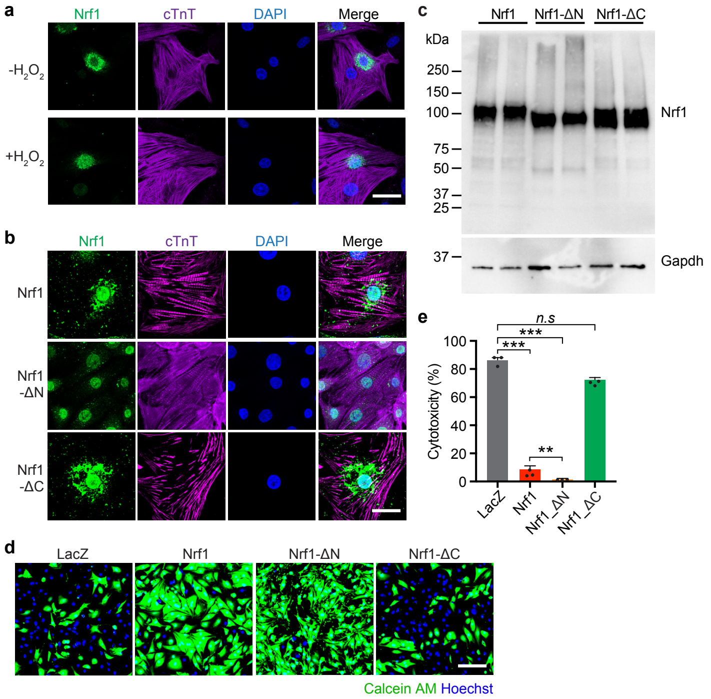

Supplementary Fig. 10. Transcriptional activity of Nrf1 is required for its cytoprotective role. a, Immunostaining on NRVMs overexpressing Nrf1 either untreated (-H_2O_2) or after treatment (+H_2O_2) with H_2O_2 showing nuclear translocation of Nrf1 in response to H_2O_2. Scale bar, 20 µm. **b**, Immunostaining of Nrf1 and cTnT to show the subcellular localization of Nrf1, Nrf1-ΔN, and Nrf1-ΔC in NRVMs. Scale bar, 20 µm. **c**, Western blot analysis showing overexpression of Nrf1, Nrf1-ΔN, or Nrf1-ΔC in NRVMs. **d, e**, Viability as measured with Calcein AM (green) (**d**) and percent of cell death (**e**) of NRVMs overexpressing LacZ, Nrf1, Nrf1-ΔN, or Nrf1-ΔC after H_2O_2 treatment; n=5 for each group. Scale bar, 100 µm. Results are shown as mean ± s.d.; *n.s.*, not significant; **p=0.0045, ***p<0.0001 by Student's t-test two-tailed.

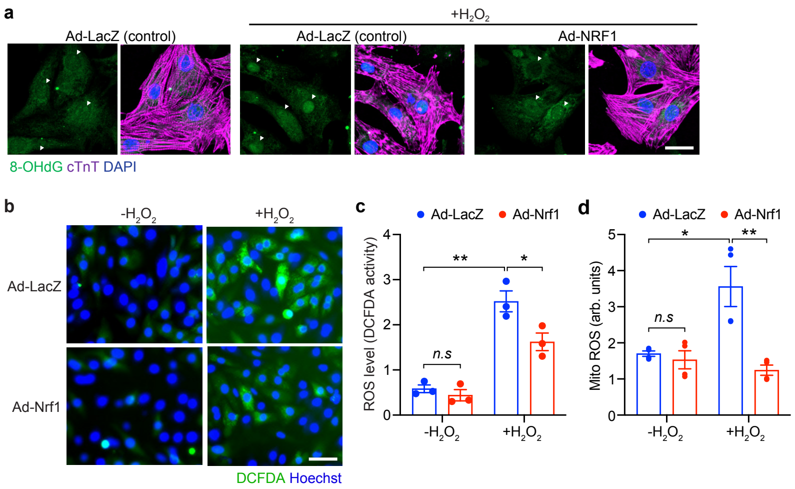

Supplementary Fig. 11. Nrf1 overexpression alleviates the oxidative stress in NRVMs treated with H_2O_2. a, Immunostaining of cTnT for cardiomyocytes and 8-OHdG for DNA damage in NRVMs overexpressing LacZ or Nrf1 after H_2O_2 treatment. Scale bar, 20 µm. Arrowheads indicate 8-OHdG activity in the nucleus. **b**, ROS activity measured by DCFDA (green) in NRVMs overexpressing LacZ or Nrf1 cells after H_2O_2 treatment. Scale bar, 50 µm. **c**, Quantification of ROS levels in NRVMs overexpressing LacZ or Nrf1 before and after H_2O_2 treatment; **p=0.0014, *p=0.0413. **d**, Quantification of mitochondrial ROS levels in NRVMs overexpressing LacZ or Nrf1 before and after H_2O_2 treatment; *p=0.0159, **p=0.0067. **c, d**, results are shown as mean ± s.e.m.; n=3 for each group, *n.s.*, not significant; Student's t-test two-tailed; arb. units, arbitrary units.

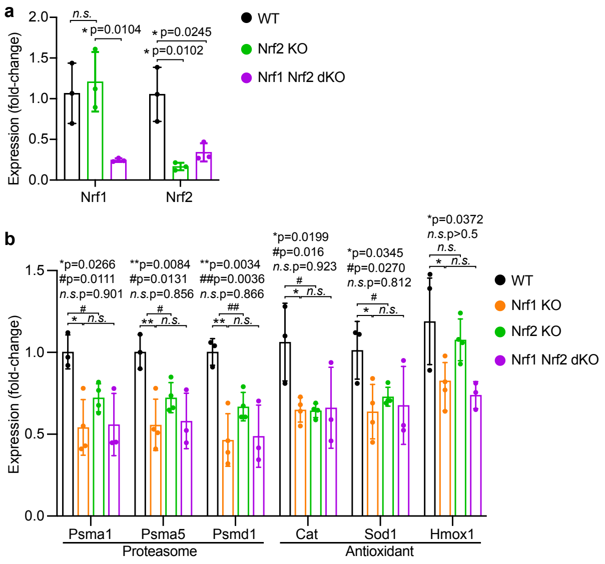

Supplementary Fig. 12. Expression of proteasome and antioxidant genes in hearts of Nrf1 cKO, Nrf2 cKO, and Nrf1/Nrf2 double knockout (dKO) mice. a, *Nrf1* and *Nrf2* expression (qPCR) in hearts from wildtype (WT), Nrf2 cKO and Nrf1/Nrf2 dKO mice at P14; n=3 for each group. **b**, qPCR measurement of proteasome subunit genes and antioxidant genes in WT, Nrf1 cKO, Nrf2 cKO, and Nrf1/Nrf2 dKO hearts at P14; n= 3 animals for groups WT and Nrf1 Nrf2 dKO; n= 4 animals for groups Nrf1 KO and Nrf2 KO. Results are shown as mean ± s.d; *n.s.*, not significant; Student's t-test two-tailed with exact p-values depicted.

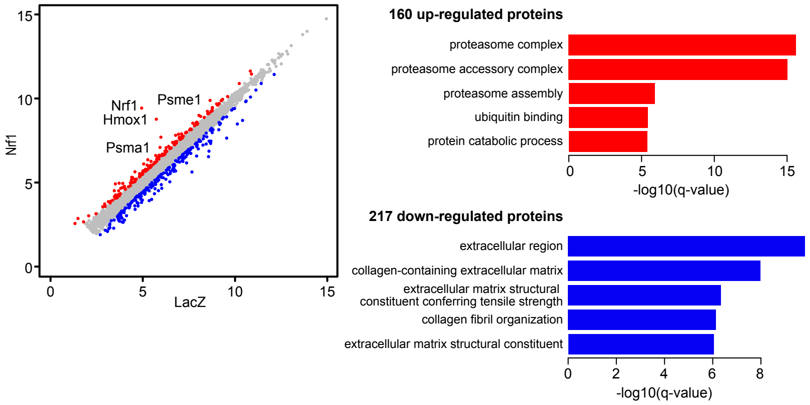

Supplementary Fig. 13. Proteomics analysis reveals increased protein levels of proteasome subunits and antioxidant enzymes in response to Nrf1 overexpression. Tandem Mass Tag proteomics quantification identifies proteins with increased (red) or decreased (blue) abundance in NRVMs overexpressing Nrf1 compared to LacZ control cells (cutoffs: fold-change > 1.5 and FDR < 0.05). Log2 reporter ion intensities are plotted (left) and GO terms associated with the up-regulated and down-regulated proteins are shown (right). n=3 for each group.

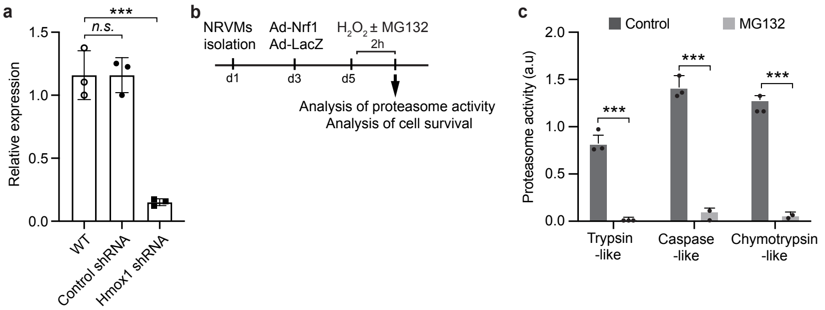

Supplementary Fig. 14. The efficacy of Hmox1 shRNA and MG132 proteasome inhibition. **a**, Relative expression of *Hmox1* transcripts in NRVMs expressing *Hmox1* shRNA or control shRNA; n= 3 biologically independent experiments for each group; ***p=0.0009. **b**, Timeline showing MG132 treatment in NRVMs. **c**, Proteasome activity from NRVMs treated with proteasome inhibitor MG132 compared to control cells; n=2 for Caspase-like and Chymotrypsin-like groups with MG132 treatment; n=3 for each of the rest groups; ***p<0.0001. **a**, **c**, results are shown as mean ± s.e.m; *n.s.*, not significant; Student's t-test two-tailed.

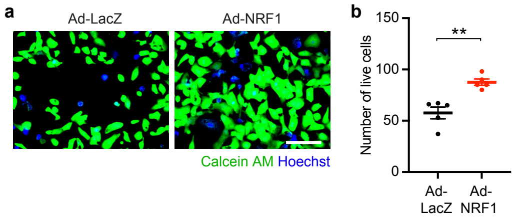

Supplementary Fig. 15. NRF1 overexpression protects human iPSC-derived cardiomyocytes from doxorubicin induced toxicity. a, Viable hPSC-CMs with adenoviral (Ad) overexpression of LacZ or NRF1 following doxorubicin treatment, indicated by Calcein AM (green); Scale bar, 100 µm. **b**, Quantification of viable cells in (**a**). Results are shown as mean ± s.e.m; n=5 for each group; **p=0.0015, by Student's t-test two-tailed.

Supplementary Table 1. qPCR primer sequences

Gene	Species	Forward primer	Reverse primer
Psma1	Mouse	GCCCGTTCTCAATCAGCTCGTAC	GCTCTGCAGGGAGTGTTTCTC
Psma5	Mouse	GCAAGTCATGGAGGAGAAGCTG	GAAGTTCGAGGACGGCTCCTTC
Psmb3	Mouse	GGTCGACAGATCAAGCCTTAC	GCAAATGAAGGGCTTGAAGGTC
Psmd1	Mouse	TCACACTTCCTGTCATTGGC	GAGGGGCAGGATATGCGAATG
Cat	Mouse	TCCAGGCTCTTCTGGACAAG	AGGCTGAGCACCGGAGTTAC
Sod1	Mouse	CACTCTCAGGAGAGCATTCCATC	GGCTCCCAGCATTTCCAGTC
Sod2	Mouse	GCAAGGTCGCTTACAGATTGC	GTAGTAAGCGTGCTCCCACACG
Hmox1	Mouse	TCACAGATGGCGTCACTTCG	GTGTCTGGGATGAGCTAGTGC
Nrf1	Mouse	GACAAGATCATCAACCTGCCTGTAG	CCGGATATCCCGGATGAGGC
Nrf2	Mouse	AAAGCACAGCCAGCACATTC	GGGATTCACGCATAGGAGCA
PSMA1	Human	AGGTTCAGCCACAGTTGGTCTG	TAAGCCCCGCAATTGAGATACC
PSMD1	Human	CCCCGTGAACTACGTGAGGC	GACTTTGGAATACAGCTGTCTGAAC
SOD1	Human	AGATGGTGTGGCCGATGTGTC	CCACCTTTGCCCAAGTCATCTGC
HMOX1	Human	AGATGACTCCCGCAGTCAGGC	TGGTCCTTGGTGTCATGGGTC
NRF1	Human	CCTTTGGGGAGAATGCTGAGT	CCGGTCAGAAGAGGAGACAAG
Hmox1	Rat	CAGAGTCCCTCACAGACAGAG	GGAGCGGTGTCTGGGATGAAC
