# Supplementary material for: Nrf1 promotes heart regeneration and repair by regulating proteostasis and redox balance
Source: Nat Commun. 2021 Sep 6;12:5270. doi: 10.1038/s41467-021-25653-w (PMC8421386; doi:10.1038/s41467-021-25653-w)
Supplement: Supplementary file 3 — Description of Additional Supplementary Files [file 41467_2021_25653_MOESM3_ESM.docx]

File Name: Supplementary Data 1

Description: Genes differentially regulated by Nrf1 deletion in CM4 cells.

File Name: Supplementary Data 2

Description: Genes differentially regulated by Nrf1 or Nrf2 overexpression.

File Name: Supplementary Data 3

Description: Proteins differentially expressed by Nrf1 overexpression measured by TMT proteomics.

File Name: Supplementary Data 4

Description: Genes correlate with NRF1 expression in 431 human heart samples from the GTEx database.
