# Supplementary material for: Nrf1 promotes heart regeneration and repair by regulating proteostasis and redox balance
Source: Nat Commun. 2021 Sep 6;12:5270. doi: 10.1038/s41467-021-25653-w (PMC8421386; doi:10.1038/s41467-021-25653-w)
Supplement: Supplementary file 8 — Reporting Summary [file 41467_2021_25653_MOESM8_ESM.pdf]

nature research

Corresponding author(s): Eric N. Olson

Last updated by author(s): Aug 5, 2021

Reporting Summary

Nature Research wishes to improve the reproducibility of the work that we publish. This form provides structure for consistency and transparency in reporting. For further information on Nature Research policies, see our Editorial Policies and the Editorial Policy Checklist.

Statistics

For all statistical analyses, confirm that the following items are present in the figure legend, table legend, main text, or Methods section.

n/a	Confirmed	
☐	☒	The exact sample size (*n*) for each experimental group/condition, given as a discrete number and unit of measurement
☐	☒	A statement on whether measurements were taken from distinct samples or whether the same sample was measured repeatedly
☐	☒	The statistical test(s) used AND whether they are one- or two-sided<br>*Only common tests should be described solely by name; describe more complex techniques in the Methods section.*
☒	☐	A description of all covariates tested
☐	☒	A description of any assumptions or corrections, such as tests of normality and adjustment for multiple comparisons
☐	☒	A full description of the statistical parameters including central tendency (e.g. means) or other basic estimates (e.g. regression coefficient) AND variation (e.g. standard deviation) or associated estimates of uncertainty (e.g. confidence intervals)
☐	☒	For null hypothesis testing, the test statistic (e.g. *F*, *t*, *r*) with confidence intervals, effect sizes, degrees of freedom and *P* value noted<br>*Give P values as exact values whenever suitable.*
☒	☐	For Bayesian analysis, information on the choice of priors and Markov chain Monte Carlo settings
☒	☐	For hierarchical and complex designs, identification of the appropriate level for tests and full reporting of outcomes
☒	☐	Estimates of effect sizes (e.g. Cohen's *d*, Pearson's *r*), indicating how they were calculated

Our web collection on statistics for biologists contains articles on many of the points above.

Software and code

Policy information about availability of computer code

Data collection	Tandem mass tag proteomics data were collected using Proteome Discoverer v2.4 (Thermofisher); sequencing data were collected using Illumina Nextseq 500 system; echo data were collected using VisualSonics Vevo2100 imaging system; Calcium imaging and Contractile force measurements data were collected using Nikon A1R+ confocal system; Confocal images were acquired using Zeiss Zen-2 or Leica LAS X.
Data analysis	Information is provided in manuscript: Methods (Tandem mass tag proteomics analysis, Spatial transcriptome data analysis, snRNA-seq Data Analysis, and Bulk RNA Sequencing in NRVMs and Data Analysis). Software used for data analysis include Space Ranger Single-Cell Software Suit (10x Genomics), Seurat R package v3.2, Cell Ranger Single-Cell Software Suit (10x Genomics), FastQC Tool (Version 0.11.4), Samtools v0.1.18 , featurecount (Version 1.6.0) , edgeR (Version 3.20.5), Fiji (ImageJ, Version 2.1.0), Clampfit 10.7 software (Axon Instrument), MAGIC (Version 3.0.0), Metascape, REVIGO.

For manuscripts utilizing custom algorithms or software that are central to the research but not yet described in published literature, software must be made available to editors and reviewers. We strongly encourage code deposition in a community repository (e.g. GitHub). See the Nature Research guidelines for submitting code & software for further information.

Data

Policy information about availability of data

All manuscripts must include a data availability statement. This statement should provide the following information, where applicable:

- Accession codes, unique identifiers, or web links for publicly available datasets
- A list of figures that have associated raw data
- A description of any restrictions on data availability

Data availability: All data presented in this study are available in the main text or the supplementary materials. Raw and analyzed RNA-sequencing data generated

during this study are available in the Gene Expression Omnibus (GEO) repository (http://www.ncbi.nlm.nih.gov/geo/) and are accessible through GEO series accession number GSE163631 [https://www.ncbi.nlm.nih.gov/geo/query/acc.cgi?acc=GSE163631]. Raw proteomics data are available in MassIVE [https://massive.ucsd.edu/ProteoSAFe/private-dataset.jsp?task=4170f66badb7404390b9a713900e733a]. Source data are provided with this paper. Previously published datasets used in this study include: GSE130699 [https://www.ncbi.nlm.nih.gov/geo/query/acc.cgi?acc=GSE130699], GSE147236 [https://www.ncbi.nlm.nih.gov/geo/query/acc.cgi?acc=GSE147236], GSE1145 [https://www.ncbi.nlm.nih.gov/geo/query/acc.cgi?acc=GSE1145], GSE9800 [https://www.ncbi.nlm.nih.gov/geo/query/acc.cgi?acc=GSE9800], GSE95755 [https://www.ncbi.nlm.nih.gov/geo/query/acc.cgi?acc=GSE95755], and GTEx [https://gtexportal.org/home/].

Field-specific reporting

Please select the one below that is the best fit for your research. If you are not sure, read the appropriate sections before making your selection.

[x] Life sciences [ ] Behavioural & social sciences [ ] Ecological, evolutionary & environmental sciences

For a reference copy of the document with all sections, see nature.com/documents/nr-reporting-summary-flat.pdf

Life sciences study design

All studies must disclose on these points even when the disclosure is negative.

Sample size	Sample sizes were not pre-determined based on statistical power calculations, but were based on our previous experience, experimental approach, and literature conducting similar experimentation. For assays in which variability is commonly high (such as animal studies), we typically used n>5. For assays in which variability is commonly low (such cell culture experiments), we typically used n<5. Sample sizes for next generation sequencing data were determined following ENCODE consortium guidelines (https://www.encodeproject.org/documents/cede0cbe-d324-4ce7-ace4-f0c3eddf5972/@@download/attachment/ENCODE%20Best%20Practices%20for%20RNA_v2.pdf).
Data exclusions	No data were excluded.
Replication	All attempts at replication for standard assays (i.e. LDH assay, live/dead cells staining assay, qPCR, immunohistochemistry, echocardiography) were successful. A majority of experiments were performed in three or more biological replicates with the exception of the Spatial Transcriptome and single nucleus RNA sequencing. However, the Spatial Transcriptome analysis of timepoint P1-D7-MI was repeated twice with biologically independent animals; samples (P1-D3-Sham, P1-D3-MI, P1-D7-Sham) were performed once due to the cost of such experiment and already observed high reproducibility between the two replicates of P1-D7-MI. For the single nucleus RNA sequencing experiment, pooled hearts (n=4) were used for each sample to account for any possible individual and dissection variabilities.
Randomization	No formal randomization techniques were used. However, samples were allocated randomly to experiments and processed in an arbitrary order.
Blinding	Investigators were blinded to group allocation during data collection and data analysis for echocardiography analyses, cardiac injury surgeries, Triphenyltetrazolium chloride staining, measurements of calcium signaling, sarcomere structure and contractile force of the iPSC-CMs. For other experiments, investigators were not blinded to group allocation during data collection due to the necessary prior knowledge of sample identity for sample pooling and collection. However, investigators were blinded to group allocation during data analysis in most of cases.

Reporting for specific materials, systems and methods

We require information from authors about some types of materials, experimental systems and methods used in many studies. Here, indicate whether each material, system or method listed is relevant to your study. If you are not sure if a list item applies to your research, read the appropriate section before selecting a response.

Materials & experimental systems

n/a	Involved in the study	
[ ]	[x]	Antibodies
[ ]	[x]	Eukaryotic cell lines
[x]	[ ]	Palaeontology and archaeology
[ ]	[x]	Animals and other organisms
[x]	[ ]	Human research participants
[x]	[ ]	Clinical data
[x]	[ ]	Dual use research of concern

Methods

n/a	Involved in the study	
[x]	[ ]	ChIP-seq
[x]	[ ]	Flow cytometry
[x]	[ ]	MRI-based neuroimaging

Antibodies

Antibodies used	cTnT (13-11) (Invitrogen, MA5-12960, 1:200)<br>pH3 (Ser10) (Cell Signaling Technology, 9701S, 1:200)<br>Nrf1 (Abcam, ab238154, 1:250)<br>CD45 (30-F11) (Tonbo Biosciences, 70-0451, 1:250)

Vimentin (280618) (R&D Systems, MAB2105, 1:250)
cTnI (Abcam, ab47003, 1:200)
8-OHdG (Novus Biologicals, NB600-1508, 1:250)
ubiquitin (P4D1) (Cell Signaling Technology, #3936, 1:5000)
Hmox1 (Proteintech, 10701-1-AP, 1:500)
Nrf1 (D5B10) (Cell Signaling Technology, #8052, 1:1000)
Nrf2 (Abcam, ab137550, 1:1000)
Goat anti-mouse IgG Alexa 647 (ThermoFisher Scientific A32728, 1:500)
Goat anti-mouse IgG Alexa 488 (ThermoFisher Scientific A32723, 1:500)
Goat anti-rabbit IgG Alexa 647 (ThermoFisher Scientific A32731, 1:500)
Goat anti-rat IgG Alexa 488 (ThermoFisher Scientific A-11006, 1:500)
Goat anti-mouse IgG HRP (abcam, ab6789, 1:1000)
Goat anti-rabbit IgG HRP (abcam, ab6721, 1:1000)
Donkey anti-mouse secondary antibody conjugated with Alexa 647 (Thermo Fisher Scientific, A32787, 1:500)
Donkey anti-goat secondary antibody conjugated with Alexa 488 (Thermo Fisher Scientific, A-11055, 1:500)

Validation

All antibodies are commercially available and have been validated in previously published studies, e.g.PNAS(PMID: 31451669) and Dev Cell PMID:33290696). We have independently validated antibodies that were central to our conclusions. For instance, the anti-Nrf1 antibody was validated using Nrf1 CKO mice.

cTnT (13-11) (Invitrogen, MA5-12960, 1:200) https://www.thermofisher.com/antibody/product/Cardiac-Troponin-T-Antibody-clone-13-11-Monoclonal/MA5-12960
pH3 (Ser10) (Cell Signaling Technology, 9701S, 1:200) https://www.cellsignal.com/products/primary-antibodies/phospho-histone-h3-ser10-antibody/9701
Nrf1 (Abcam, ab238154, 1:250) https://www.abcam.com/nfe2l1-antibody-ab238154.html
CD45 (30-F11) (Tonbo Biosciences, 70-0451, 1:250) https://tonbobio.com/products/purified-anti-mouse-cd45-30-f11
Vimentin (280618) (R&D Systems, MAB2105, 1:250) https://www.rndsystems.com/products/human-mouse-rat-vimentin-antibody-280618_mab2105
cTnI (Abcam, ab47003, 1:200) https://www.abcam.com/cardiac-troponin-i-antibody-ab47003.html
8-OHdG (Novus Biologicals, NB600-1508, 1:250) https://www.novusbio.com/products/8-ohdg-antibody_nb600-1508
ubiquitin (P4D1) (Cell Signaling Technology, #3936, 1:5000) https://www.cellsignal.com/products/primary-antibodies/ubiquitin-p4d1-mouse-mab/3936
Hmox1 (Proteintech, 10701-1-AP, 1:500) https://www.ptglab.com/products/HMOX1-Antibody-10701-1-AP.htm
Nrf1 (D5B10) (Cell Signaling Technology, #8052, 1:1000) https://www.cellsignal.com/products/primary-antibodies/tcf11-nrf1-d5b10-rabbit-mab/8052
Nrf2 (Abcam, ab137550, 1:1000) https://www.abcam.com/nrf2-antibody-ab137550.html
Goat anti-mouse IgG Alexa 647 (ThermoFisher Scientific A32728, 1:500) https://www.thermofisher.com/antibody/product/Goat-anti-Mouse-IgG-H-L-Highly-Cross-Adsorbed-Secondary-Antibody-Polyclonal/A32728
Goat anti-mouse IgG Alexa 488 (ThermoFisher Scientific A32723, 1:500) https://www.thermofisher.com/antibody/product/Goat-anti-Mouse-IgG-H-L-Highly-Cross-Adsorbed-Secondary-Antibody-Polyclonal/A32723
Goat anti-rabbit IgG Alexa 647 (ThermoFisher Scientific A32731, 1:500) https://www.thermofisher.com/antibody/product/Goat-anti-Rabbit-IgG-H-L-Highly-Cross-Adsorbed-Secondary-Antibody-Polyclonal/A32731
Goat anti-rat IgG Alexa 488 (ThermoFisher Scientific A-11006, 1:500) https://www.thermofisher.com/antibody/product/Goat-anti-Rat-IgG-H-L-Cross-Adsorbed-Secondary-Antibody-Polyclonal/A-11006
Goat anti-mouse IgG HRP (abcam, ab6789, 1:1000) https://www.abcam.com/goat-mouse-igg-hl-hrp-ab6789.html
Goat anti-rabbit IgG HRP (abcam, ab6721, 1:1000) https://www.abcam.com/goat-rabbit-igg-hl-hrp-ab6721.html
Donkey anti-mouse secondary antibody conjugated with Alexa 647 (Thermo Fisher Scientific, A32787, 1:500) https://www.thermofisher.com/antibody/product/Donkey-anti-Mouse-IgG-H-L-Highly-Cross-Adsorbed-Secondary-Antibody-Polyclonal/A32787
Donkey anti-goat secondary antibody conjugated with Alexa 488 (Thermo Fisher Scientific, A-11055, 1:500) https://www.thermofisher.com/antibody/product/Donkey-anti-Goat-IgG-H-L-Cross-Adsorbed-Secondary-Antibody-Polyclonal/A-11055

Eukaryotic cell lines

Policy information about cell lines

Cell line source(s)

Adeno-X 293 (Clontech, 632267). Primary cardiomyocytes isolated from 1- or 2-day-old Sprague-Dawley rats using the Isolation System for Neonatal Rat/Mouse Cardiomyocytes (Cellutron, nc-6031). hPSCs were derived from a healthy donor.

Authentication

Adeno-X 293 cells were not authenticated in this study.

Mycoplasma contamination

All cell lines tested negative for mycoplasma contamination.

Commonly misidentified lines
(See ICLAC register)

No commonly misidentified cell lines were used.

Animals and other organisms

Policy information about studies involving animals; ARRIVE guidelines recommended for reporting animal research

Laboratory animals	All mouse experiments complied with all relevant ethical regulations and were performed according to protocols approved by the Institutional Animal Care and Use Committees at University of Texas Southwestern Medical Center. UT Southwestern uses the "Guide for the Care and Use of Laboratory Animals" when establishing animal research standards. All mice used in this study were housed at the pathogen-free Animal Resource Center at the University of Texas Southwestern Medical Center. All animals were bred inside a SPF facility with 12h light/dark cycles with a temperature of 18–24 °C and humidity of 35–60% and monitored daily with no health problems. All animals were housed in groups of maximum five per cage with ad libitum access to food and water. Nrf2 fl/fl mice were obtained from the Jackson Laboratory (# 025433).<br>All animals, including Nrf1fl/fl:aMHC-Cre, Nrf2fl/fl:aMHC-Cre, Nrf1fl/fl:Nrf2fl/fl:aMHC-Cre, and wildtype mice, used in this study are in C57/Bl6 background. Both female and male animals were used for all experiments, except for the Ischemia /reperfusion experiment where only male animals were used. The reason for this is that females animals have better cardio-protection than males (Ref: https://academic.oup.com/cardiovascres/article/75/3/478/347648). Both neonatal stages (P1-P8) and the adult stage (6-8wks) were used in this study with the specific stage for each experiment described in Methods.
Wild animals	The study did not involve wild animals.
Field-collected samples	The study did not involve field-collected samples.
Ethics oversight	All experiments involving animals were approved by the Institutional Animal Care and Use Committee at the University of Texas Southwestern Medical Center.

Note that full information on the approval of the study protocol must also be provided in the manuscript.
